# Quadrant-Based Minimum Bounding Rectangle-Tree Indexing Method for Similarity Queries over Big Spatial Data in HBase

**DOI:** 10.3390/s18093032

**Published:** 2018-09-10

**Authors:** Bumjoon Jo, Sungwon Jung

**Affiliations:** Department of Computer Science and Engineering, Sogang University, 35 Baekbeom-ro, Mapo-gu, Seoul 04107, Korea; bumjoonjo@sogang.ac.kr

**Keywords:** HBase, NoSQL, big spatial data, spatial data indexing, QbMBR tree, range query, kNN query

## Abstract

With the rapid development of mobile devices and sensors, effective searching methods for big spatial data have recently received a significant amount of attention. Owing to their large size, many applications typically store recently generated spatial data in NoSQL databases such as HBase. As the index of HBase only supports a one-dimensional row keys, the spatial data is commonly enumerated using linearization techniques. However, the linearization techniques cannot completely guarantee the spatial proximity of data. Therefore, several studies have attempted to reduce false positives in spatial query processing by implementing a multi-dimensional indexing layer. In this paper, we propose a hierarchical indexing structure called a quadrant-based minimum bounding rectangle (QbMBR) tree for effective spatial query processing in HBase. In our method, spatial objects are grouped more precisely by using QbMBR and are indexed based on QbMBR. The QbMBR tree not only provides more selective query processing, but also reduces the storage space required for indexing. Based on the QbMBR tree index, two query-processing algorithms for range query and kNN query are also proposed in this paper. The algorithms significantly reduce query execution times by prefetching the necessary index nodes into memory while traversing the QbMBR tree. Experimental analysis demonstrates that our method significantly outperforms existing methods.

## 1. Introduction

Due to the development of data acquisition techniques, the main sources of modern spatial data have become sensors, the Internet of Things (IoT), social media, and mobile phones. As a result of these environmental changes, handling large-sized geo-tagged data has become one of the important issues of the current data management systems. Although traditional spatial data such as satellite imagery, road networks, and raster images also have been classified as large data, the sizes of the geo-spatial datasets generated in the Internet of Everything (IoE) environment exceed the capacity of the current computing systems [1]. These data are currently in the order of PB, and their size is steadily increasing [1,2]. Therefore, in recent years, a significant number of methods for dealing with large-volume spatial data in a distributed storage environment have been developed.

In order to efficiently store and search big spatial data, many studies have attempted to use the Hadoop distributed file system and the MapReduce technique [3,4,5]. However, these methods suffer from a large number of disk I/O operations during spatial query processing because of a lack of spatial awareness of the underlying file systems. SpatialHadoop [6] and Hadoop-GIS [7] were developed to enhance the spatial awareness of the underlying distributed file systems. They modify the core of the underlying file system to group spatial data according to their spatial proximity and distribute them across multiple servers. However, these methods are not effective for frequently updated spatial data because their data distribution policy and indexes are primarily designed for static data. When a large amount of the data update occurs, these methods should investigate the distribution of data again and reconstruct indexes, which is a very costly process.

**Figure 1:**
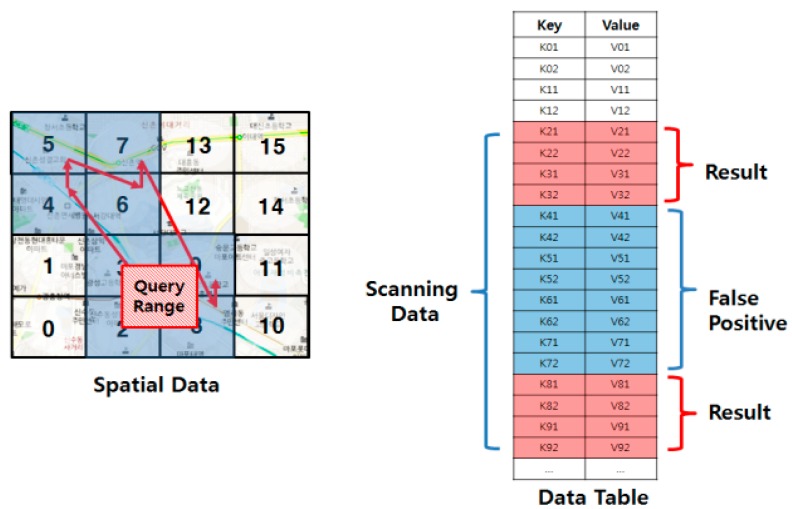
Range query processing using a linearization technique.

HBase and linearization techniques [8,9] can be a good alternative solution for this problem. HBase is one of the most widely used NoSQL databases, which provides an effective framework for fast random data access and efficient data updates in a distributed file system. As HBase sorts data in a lexicographic order of row key and horizontally divides the table to distribute data among servers, the linearization techniques allow us to easily partition data according to their spatial proximity. Figure 1 presents an example of range query processing based on a linearization technique. The range query finds a set of data points whose distance from the query point is less than a given query range. When a range query is issued, the minimum and the maximum z-order values within the query range are calculated. The query is then processed by scanning the rows whose row keys are within the z-order range.

As the linearization techniques cannot completely guarantee the spatial proximity of data, the linearization-based approaches sometimes include false positives, as shown in Figure 1. Therefore, a significant number of studies [10,11,12,13,14,15] have recently proposed to reduce the false positives of query processing by implementing a secondary index on top of HBase. These methods use the multi-dimensional index such as a kd-tree or an R-tree to determine more accurately the range of the row key. The problem of these methods is the granularity of the index. Due to the storage overhead of this index and its search time, these methods partition and index the data into large units, such as HBase region sizes. Moreover, the search process of these methods may investigate unrelated regions because they roughly partition the data using fixed sized grid cells.

To solve this problem, we previously proposed an indexing method [14] using quadrant-based MBR (QbMBR). The key idea of this method is that it dynamically divides the space into quadrants, and represents the distribution of data in each quadrant by a minimum bounding rectangle (MBR). The quadrant-based MBRs are indexed by using a hierarchical index structure similar to a quad-tree. Since our index dynamically partitions data into a smaller unit, it can support more selective query processing with less storage overhead. In this paper, we propose improved algorithms for range query processing and index update, as well as a new kNN query-processing algorithm. The contributions of this paper may be summarized as follows:

- We present analyses for the storage overhead of our QbMBR tree. In our analyses, QbMBR tree indexing not only improves query processing performance under various conditions, but also requires less storage overhead than other methods.
- We propose improved query processing algorithms. In HBase, frequent RPCs cause performance degradation for searching hierarchical indexes. The performances of our insertion and range query processing algorithms are enhanced with fewer RPCs.
- We propose a new query processing algorithm for a kNN query. We use an approximate kNN range to determine the minimum range that should contain all of the candidate objects.
- We evaluate the performance of proposed algorithms using various datasets. Two kinds of synthetic datasets (with uniform and normal distribution) and two real datasets (OpenStreetMap and European POI dataset) are used to evaluate our query processing algorithms. Experimental results show that our algorithms demonstrate superior performance for both cases of synthetic and real datasets.

This paper is organized as follows: in Section 2, we discuss related works. In Section 3, we describe our index structure—the QbMBR tree—and the analysis of its storage overhead. Section 4 describes the query processing algorithms for insertion, range, and k-NN queries, which use the QbMBR tree. In Section 5, we experimentally evaluate the performance of our indexing structure and algorithms. Section 6 contains our final conclusions.

## 2. Related Works

The linearization technique is used to generate sequential row keys for spatial objects in HBase. This method subdivides the data space into grid-shaped cells, and sequentially orders the cells using space-filling curves. The row keys for the spatial objects are produced by using the geo-hashing number of the corresponding cells. However, since the linearization techniques cannot completely guarantee the spatial proximity of data, a significant amount of studies has attempted to reduce the false positives of the linearization technique in various ways. These methods can be categorized into three types according to their implementation layer.

The first approach is to modify the core of the HBase system, especially the file access method, to support the more selective spatial query processing. Pyro [16] modifies the cores of Hadoop and HBase to support the fine-grained spatial query processing from the system level. This method optimizes the file access strategy to provide more selective data fetching, and imposes a block replica placement policy to support load balancing of region servers. STEHIX [17] modifies the index architecture of HBase to implement a local multi-dimensional index for the StoreFile in the region server. However, these methods are difficult to extend because they are dedicated systems for handling spatio-temporal data. In addition, system level modification is very costly and complex when comparing with the other approaches.

The second approach is to construct an in-memory index layer in the client of the HBase system. R-HBase [10] firstly divides the data space into fixed sized grid cells, and builds an R-tree in memory to index the cells. The objects located within the same cells are grouped together and are indexed with the same leaf node. BGRP-tree [11] also uses an in-memory R-tree. In this method, objects are indexed by unit of Hbase region, and the additional index named BGRP-tree is maintained to manage the result of dynamic region splitting. The limitation of these approaches is that the leaf node covers a wide area of space and each leaf node contains many objects. For example, the leaf node of the BGRP-tree covers all objects located within a single region. Since there is a tradeoff between the granularity and storage overhead of the index, an index for higher resolution is hard to implement on a client memory. Moreover, the fixed sized grid cells used in these methods cannot represent the actual distribution of objects.

**Figure 2:**
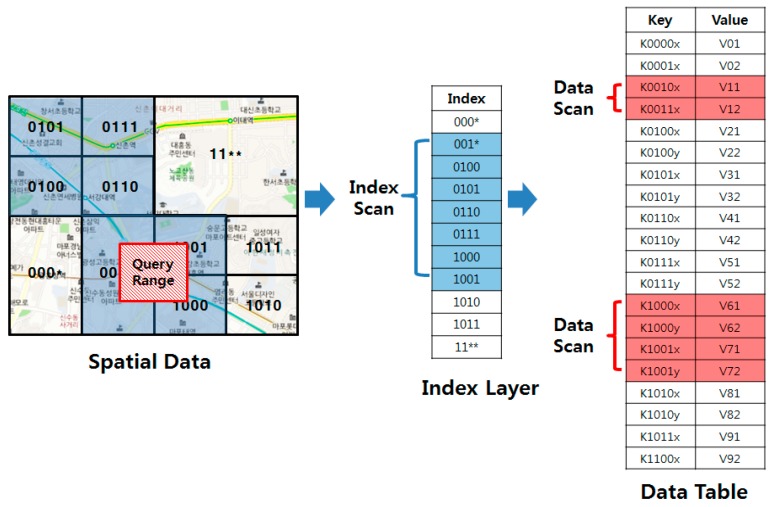
Range query processing using an indexing layer.

The final approach implements a multi-dimensional index in the form of an HBase data table. The multi-dimensional index table is firstly scanned to identify the row keys relevant to the query range. For example, MD-HBase [12] first enumerates the objects using z-order, and partitions the objects based on a kd-tree or a quad-tree. After partitioning, the longest common prefix of the object in the same partitioned area is stored in an index table. Figure 2 shows an example of range query processing using a multi-dimensional index in MD-HBase. When a range query is issued, the indexing layer is searched first, and only the rows that have row keys associated with relevant regions are transmitted to the client. KR+ indexing [13] groups spatial objects using an R+ tree, and the objects of the leaf nodes are stored into a single row of the table. The index table of the KR+ index contains the z-order value of a fixed-size grid cell and the row keys of the leaf nodes that are overlapped by the grid cell. The index layer-based method is suitable for indexing large amount of spatial data without complex system level modification. However, the indexes of these methods cannot support the high-resolution spatial queries, and also cannot precisely capture the actual distribution of spatial objects because they roughly group them by using space-partitioning techniques. Therefore, search processes may often investigate non-relevant areas, including a large amount of dead space that overlaps the query range.

## 3. Spatial Data Indexing Using Quadrant-Based MBR

### 3.1. Data Partitioning with Quadrant-Based MBR

**Figure 3:**
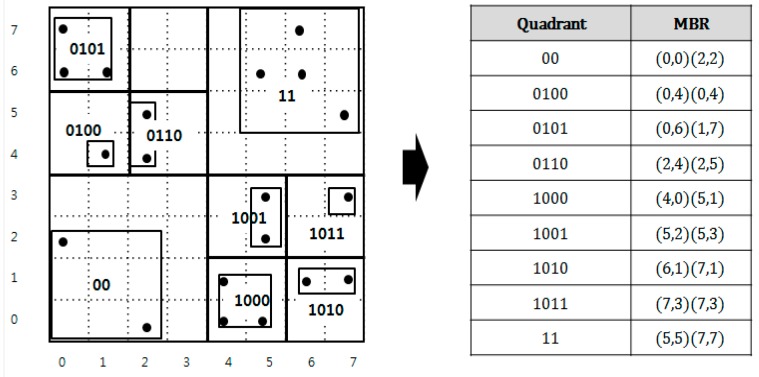
An example of QbMBR creation.

A QbMBR is an MBR consisting of objects in split quadratic space [14]. To construct the QbMBRs, we recursively divide the space into quadrants if the number of spatial objects in a quadrant exceeds a split threshold. After partitioning the space, we create an MBR for the spatial objects in each quadrant to capture more precisely the distribution of data. Figure 3 presents an example of a QbMBR for a given data space and a list of QbMBRs. In this example, we assume that the capacity of the QbMBR is four. Each quadrant is represented with bold line and the MBR in the quadrant by using two coordinates of the low-left corner and the upper-right points of a rectangle. Note that this partitioning method can create an MBR containing only a single spatial object. As shown in Figure 3, a QbMBR contains both information about the quadrant and the MBR. In our method, the quadrant information is used to identify the node, and the MBR information is used to compute the distance between the query point and the node. The reason for maintaining both of the information is to reduce update cost of our index tree. Since the quadrant is less sensitive to updated than the MBR, the row keys generated by the quadrant information allows us to avoid frequent re-insertion.

QbMBRs are stored in an HBase table, and used as building blocks of our hierarchical indexing structure. When a QbMBR is stored in an HBase table, the partitioned quadrant can be represented as a bit string, and this bit string is used as an identifier (row key) of the MBR and objects. The bit string of a quadrant is calculated in a way similar to geo hashing. The space is divided by two for each axis, and the subspace is enumerated using binary numbers. The bit string of each sub-quadrant is then obtained by interleaving the binary values of each axis. If a quadrant is recursively partitioned, then the names of the sub-quadrants are created by concatenating the name of the parent quadrant and the newly created two-bit binary string.

**Figure 4:**
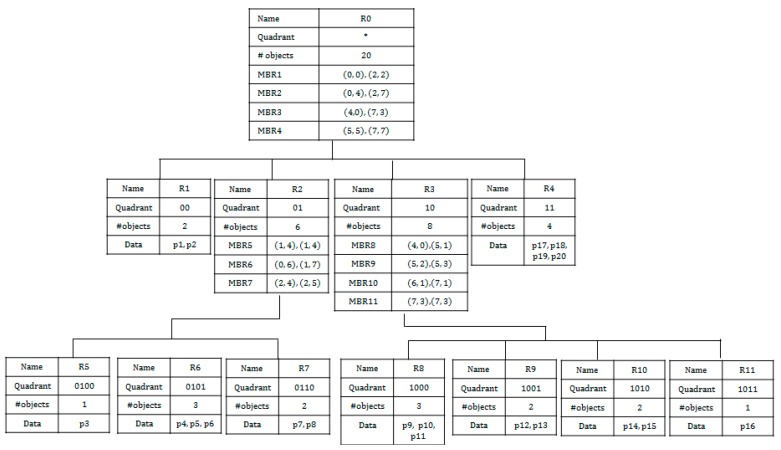
An example of a QbMBR tree.

Based on the QbMBR grouping method, we construct a hierarchical indexing structure, called the QbMBR tree, as a secondary index for spatial queries. The structure of the QbMBR tree is similar to that of a quad-tree. The main advantages of QbMBR are as follows. First, it provides a method for grouping spatial objects into smaller units when compared to the standard region splitting method in HBase. Second, the QbMBR tree does not require an additional indexing structure, unlike a BGRP tree or KR+ indexing. Therefore, the QbMBR tree method has less storage overhead. The structure of the example QbMBR tree in Figure 3 is shown in Figure 4.

### 3.2. Table Schema for Hierarchical Index on HBase

**Figure 5:**
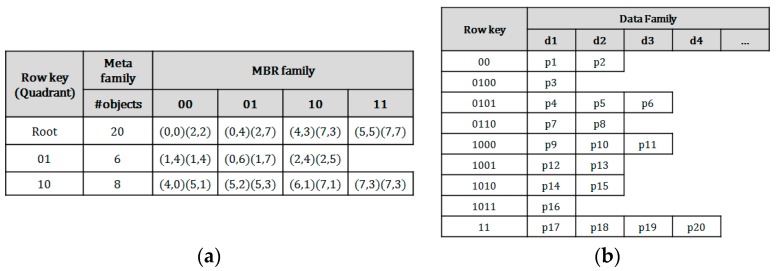
Table schema for a QbMBR tree and data objects: (**a**) An example of a row in a QbMBR tree index table; (**b**) An example of a row in a data table.

In order to provide the higher resolution index for efficient query processing in HBase, a QbMBR tree is implemented in an HBase table. The two tables are required to implement our method. The first one is an index table. The table schemata for QbMBR tree is shown in Figure 5. An internal node in Figure 5a consists of the quadrant information, the MBRs of the child nodes, and the number of objects included in their sub-trees. As mentioned in Section 3.1, the bit-string of the quadrant is used as the row key of each node, and the MBR information of each node is stored in a column cell, which is inexpensive to update.

In our method, the objects of the leaf nodes are stored into a single row for faster query processing. Due to the flexibility in schemas of HBase, a table of HBase can take one of two forms: tall-narrow and flat-wide. A tall-narrow table has a large number of rows with few columns, and a flat-wide table consists of a small number of rows with many columns. The format of wide table is more appropriate for loading a large-sized leaf node from the HBase table. Because the time for fetching a single row with many columns is faster than the time for fetching wide ranged rows, this table schema allows us to more promptly and selectively access the grouped spatial objects. Figure 5b represents the data table, which uses the quadrant identifiers of the corresponding leaf nodes as row keys, while storing spatial objects in its columns.

**Figure 6:**
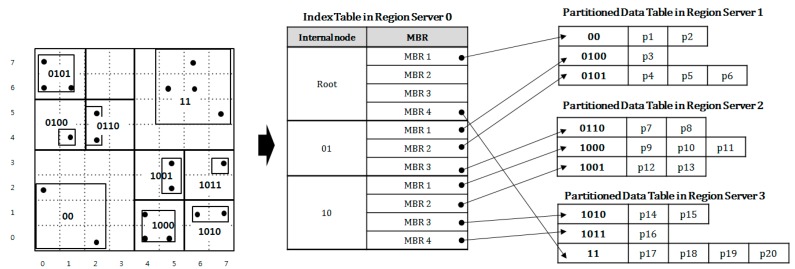
QbMBR tree index implementation on HBase.

Figure 6 shows an implementation diagram of QbMBR tree given in Figure 5 on HBase. The data table is divided into three partitions in this example. In the diagram, the three partitioned data tables are stored in the three different region servers 1 to 3 while an index table is stored in region server 0. When processing a spatial similarity query, HBase client first reads the row of the root node from the index table. Based on the MBR information of the root node, the row keys for the child nodes associated with query region are calculated. Then, the client iteratively searches the index table until it selects all the rows in which the associated objects are stored. When the search for the index table is finished, the client requests all the selected rows to the relevant region servers.

### 3.3. Storage Overhead for a QbMBR Tree

A QbMBR tree requires a smaller amount of storage overhead than a KR index or BGRP index because it does not require an additional indexing structure to manage itself. However, compared to the flat indexing structure of multi-dimensional HBase (MD-HBase), the storage requirements of a QbMBR tree are larger because the indexing structure must store information about internal nodes, as well as MBR information. If indices are created based on the same split threshold, the number of rows required to store the QbMBR tree index is approximately two to three times larger than that required for MD-HBase. However, in terms of total storage overhead, our indexing method occupies less space because the row keys of each row are generated by dynamic splitting. Unlike our methods, other methods based on linearization techniques require much higher-order z-order values to store spatial objects in order to obtain a fine-grained subspace without any knowledge about the actual data distribution.

The size of an HBase table is significantly affected by the length of the row key used because HBase stores tables in the form of key-value pairs. Specifically, HBase creates a key using a row key, column family, and column qualifier to store each column value. For example, suppose that a two-dimensional spatial object can be represented by 8 bytes. Then, the z-order value for uniquely identifying the object is 8 bytes, and the row key associated with the z-order value is also 8 bytes. For the sake of simplicity, assume that the column family and column identifier are each 1 byte. Then, the length of the entire key for storing the object is 10 bytes. If the spatial object consists of three values, such as an id, x, and y coordinates, and each is 4 bytes, then the total number of bytes required to store the spatial object is 14 + 14 + 14 = 42 bytes. However, because the grouped spatial objects in our method share a short row key, typically 4 bytes, the number of bytes required to store an object is 10 + 10 + 10 = 30 bytes. The exact number of bytes can vary depending on the schema definition of the table, but a longer row key requires more storage overhead in all cases.

**Figure 7:**
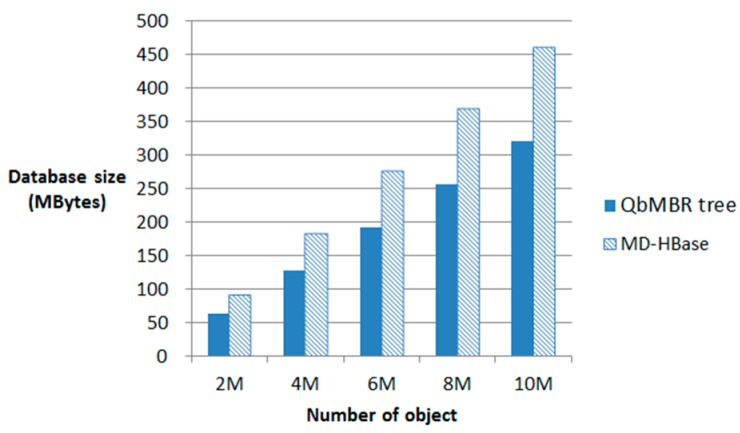
Size of a database.

Figure 7 illustrates the storage overhead for MD-HBase and QbMBR tree databases constructed using the same split threshold. Although the size of our index table is larger than the index table for MD-HBase, the size of the index table is relatively small compared to the size of the data table, and increases logarithmically as the number of objects in the database increases. Because the overhead for the longer row keys in MD-HBase increases linearly with the amount of data, the overall size of the database is 30% larger than in our method.

## 4. Algorithms for Spatial Data Insertion and Range Query Processing

### 4.1. Insertion Algorithm for a QbMBR Tree

The index search process for insertion consists of two tasks. The first task is to find the nodes of the QbMBR tree that cover the data object, and the second task is to update the nodes if necessary. In order to improve the performance of the insertion algorithm, we should minimize the number of RPCs that occur in each task. To achieve this goal, we first precompute the unit length (such as 1 byte) binary z-order value for the inserted object. Then we iteratively delete the last 2 bits of the of binary z-order value to find a set of row keys of the nodes that should be updated. If there is no leaf node among the nodes of the key set, an additional row key set is calculated and prefetched again.

Algorithm 1 presents the insertion algorithm for a QbMBR tree. In order to implement the idea for batch processing, two sets named *N* and *K* are used. *N* is a set of nodes that load in each iteration, and *K* is a set of row keys for prefetching nodes in the next iteration. The algorithm requests the rows of nodes whose row keys are stored in *K*, and stores the result in *N*. After prefetching relevant nodes, the algorithm updates the MBR of nodes if it is necessary, and inserts the object if the current node under examination is a leaf node. If there is no leaf node in the prefetched set, the additional row key set is calculated and requested again. When the number of spatial objects in a leaf node exceeds the split threshold, the function SplitNode(node) is called to split the leaf node. The function SplitNode(node), shown in Algorithm 2, returns an internal node as the result of partitioning. The splitting process creates new children by dividing a quadrant into four sub-quadrants, and redistributing the spatial objects into the newly-created leaf nodes.

**Algorithm 1.** Insert data point into a QbMBR tree
**Input:** Spatial object ***p***, split threshold ***S*** **Output:** Updated QbMBR tree after insertion ***K***←∅ ***N***←∅ bit string ***s*** = *calculate unit length geohashing value of*
***p*** **while**(***p*** is not inserted into leaf node) **do**  **while**(size of ***K*** < (*unit_length/2*)) **do**    ***K*** = ***K*** ∪ ***s***    ***s*** = delete last 2-bit of ***s***  ***N*** = prefetch nodes in ***K***  **for**(each node ***n*** in ***N***) **do**    **if**(***n*** is not a leaf node) **then**     update corresponding MBR of ***n***     increase object counter of ***n***    **else**     add spatial object ***p*** to ***n***     increase object counter of ***n***     **if**(size of ***n*** exceeds ***S***) **then**      ***n*** = ***SplitNode***(***n***)  ***s*** = *calculate additional unit length geohashing value of **p*** **End**

**Algorithm 2.** Split a node of a QbMBR tree
**Input:** A leaf node ***n*** of the QbMBR tree
**Output:** An internal node ***n*** of the QbMBR tree Create new children by dividing quadrant of ***n*** **for**(each spatial object ***p_i_*** of ***n***) **do**  **for**(each child ***c_i_*** of ***n***) **do**   **if**(quadrant of ***c_i_*** covers ***p_i_***)    add object ***p_i_*** to ***c_i_***    update MBR of ***c_i_*** stored in ***n***    increase object counter of ***c_i_*** **return**
***N***

**Figure 8:**
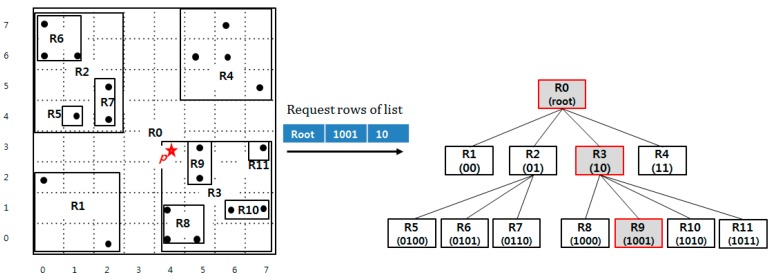
Example insertion operation.

Figure 8 presents an example of insertion. Suppose that the spatial object *p*, marked with a star in the figure, is inserted in the QbMBR tree. The algorithm first calculates the z-order value of *p*. Because the space of the example is small, we assume that the unit length of the pre-computed value is 4 bits. Therefore, the pre-computed z-order value of *p* is 1001. We then compute the row keys of relevant nodes as 1001, 10, and root. The next step is prefetching nodes in the key set. Three nodes named R0, R3, and R9 are loaded together, and their MBR is updated. Because the node R9 is a leaf node, the object *p* is inserted into R9.

### 4.2. Range Query Algorithm for a QbMBR Tree

A range query receives a query point *q* and query radius *r* as inputs and returns the set of data points whose distance from the query point is less than *r*. In order to reduce the number of RPCs during query processing, we should avoid searching the lower level nodes that cover a wide region. Therefore, the algorithm first finds the minimum-sized quadrant that covers the query range. Similar to the insertion algorithm, this process can be done by pre-computing. In order to compute the quadrant identifier, we first calculate the binary geohashing value for the lower-left and upper-right coordinates of the query region. Then the longest common prefix of these geohashing values becomes the identifier of the quadrant to start searching.

Algorithm 3 presents the range query processing algorithm with the QbMBR tree. After finding the starting node, the algorithm explores the QbMBR tree in order of breadth-first-search order. Three sets, called *N*, *K*, and *D* are used for index searching. *N* stores the nodes that are loaded for traversal in the current iteration. *K* is the set of row keys to be loaded from the index table in the next iteration. *D* is the set of row keys to be loaded from the data table. Our range query processing algorithm is presented in Algorithm 3.

The algorithm begins by inserting the row key of the root node and candidate nodes into *K*, and then loads its nodes from the index table. If the current node is an internal node, then the algorithm calculates the minimum distance between the MBRs of its children and the query point *q*. The row keys of the children whose distances are within radius *r* are inserted into *K*. After all the nodes in *N* have been traversed, the algorithm prefetches additional nodes using the row keys stored in *K* and stores the results in *N* for the next iteration. When the index searching process reaches a leaf node, the algorithm stores the row keys of the leaf node into the set *D* to load the relevant spatial objects during the final stage of the algorithm. If there are no nodes left in set *K*, the algorithm loads the spatial objects stored in the leaf nodes in *D*. The algorithm then calculates the distance between the spatial objects and the query point to answer the query. Only objects whose distances from the query point q are less than or equal to the query radius r are inserted into the result set *R*.

**Algorithm 3.** Range query algorithm**Input:** Query point ***q*** and range ***r*** **Output:** A set of spatial objects within a given query range ***N***←∅, ***D***←∅, ***K***←∅, Result set ***R***←∅ insert row key of root node into ***K*** ***q_min_*** = *unit length geohashing value of* (***q*·*x*** − ***r*, *q*·*y*** − ***r***) ***q*_*max*_** = *unit length geohashing value of* (***q*·*x* + *r*, *q*·*y* + *r***) bit string ***s*** = *longest common prefix between **q_min_** and **q_max_*** **while**(length of ***s*** > 0)  ***K** = **K*** ∪ {***s***}  ***s*** = delete last 2-bit of ***s*** ***N*** = {prefetch nodes in ***K***} ***n*_*max*_** = *a node of maximum level among **N*** **for**(*each child **n_i_** of **n**_**max**_*) **do**  **if**(***n*** is a leaf node) **then**   ***D* = *D*** ∪ {row key of ***n***}  **else if**(***n*** is not a leaf node) **then**   **for**(each MBR ***m_i_*** in ***n***) **do**    **if**(**overlap**(***m_i_***, ***q***, ***r***)) **then**     calculate row key for child and insert it into ***K*** **while**(***K*** is not empty)**do**  ***N =*** {prefetch nodes in ***K***}  **for**(each node ***n*** ∈ ***N***) **do**   **if**(***n*** is a leaf node) **then**    ***D* = *D*** ∪ {row key of ***n***}   **else if**(***n*** is not a leaf node) **then**    **for**(each MBR ***m_i_*** in ***n***) **do**     **if**(**overlap**(***m_i_***, ***q***, ***r***)) **then**      calculate row key for child and insert it into ***K*** ***P =*** {Prefetch nodes in ***D***} **for** (each object ***p*** ∈ ***P***) **do**  **if**(**distance**(***p***,***q***) <= ***r***) **then**   ***R*** = ***R*** ∪ {***p***} **Return**
***R***

**Figure 9:**
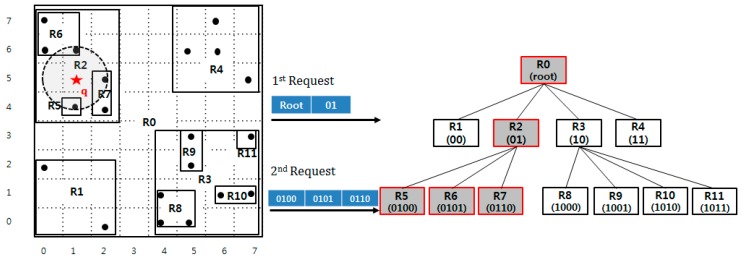
Example range query.

Figure 9 presents an example range query. The algorithm begins by pre-computing the row key of the starting node. The minimum and maximum coordinates of the query region are 1000 and 1011. Then, the bit string 10 that is the longest common prefix of 1000 and 1011 is inserted into the row key list for prefetching. The quadrant R2 exists in index and it has three children, R5, R6, and R7, which overlap the query range. The row keys R5, R6, and R7 are inserted into the key list and loaded together from the index table. Because they are leaf nodes, the algorithm inspects the data objects in each node to answer the query. Because each node contains point covered by the query area, three points are inserted into a result set *R*, and the algorithm is terminated because there are no more nodes to traverse.

### 4.3. kNN Query Processing Algorithm

A kNN query is a query that finds the *k* nearest neighbors to a query point *q*. Similar to Algorithms 1 and 3, our kNN algorithm is designed to reduce RPCs to region servers. Because the incremental range search methods commonly increase RPCs in a system, we define an approximate range *r* for loading objects. The approximate range *r* is a minimum distance that guarantees the number of sufficient spatial objects to find the desired number of neighbors. During search processing, explored nodes are sorted in order of their minimum distance, and the algorithm sequentially counts the number of objects in the nodes. If the number of objects exceeds *k*, then *r* is set to the maximum distance of the current node. The algorithm maintains two priority queues: *N* and *Q*. *N* stores nodes in ascending order of the minimum distance from the query point to their MBRs. It is used to store nodes for search planning. *Q* stores the candidate set of kNNs in descending order by distance.

Algorithm 4 presents the kNN query processing algorithm. Our algorithm consists of two phases. The first phase explores the QbMBR tree to find the row keys of leaf nodes using the approximate range. The index searching process sequentially scans the nodes stored in *N* to determine if they have a child node whose minimum distance is less than *r*. If the minimum distance of a child node is less than *r*, then the row key of the child node is inserted into list *I*. The range *r* is set very large in the initial stage of the algorithm and updated to the maximum distance of nodes in *N* if the number of objects in the nodes that are closer than the current node exceeds *k*. If the current node is a leaf node, then the algorithm stores the row key of the current node in list *L* to load them together. If there are no more nodes in *N*, the algorithm loads nodes whose row key is stored in *I*. The first phase ends when there are no more nodes in *N* or row keys in *I*. Following index searching, the algorithm loads the spatial objects of the leaf nodes stored in *L* and evaluates their distances to identify the result set of the query. The algorithm stores evaluated objects in *Q* and returns the answer set when there are no more objects to be evaluated.

Consider an example of a 2NN query. Assume that the data set and query point *q* are the same as those shown in Figure 9. The algorithm begins by examining the root node. All of the children of the root node are loaded and inserted into a priority queue in the order R2, R1, R4, R3, and *r* is set to maxdist(*q*, R2). R3 and R4 are pruned in this iteration because the minimum distances to the nodes exceed the current *r*. The algorithm then sequentially scans the nodes, and the row keys of R5, R6, and R7 are inserted into *I*, and the objects of R1 are inserted into *Q*. Because the nodes R5, R6, and R7 are still in range *r*, all of their objects are loaded at the next iteration, and the nearest two points among them are chosen as the result set.

**Algorithm 4.** kNN query algorithm **Input:** Query point ***q*** and the number of nearest neighbors, ***k*** **Output: *k*** nearest neighbors to ***q*** ***I***←∅, ***L***←∅, ***r*** = ∞ insert row key of root node into ***I*** /*explore QbMBR tree to find approximate range ***r***/ **while *I*** is not empty **do**  ***ObjectCounter*** = 0  Prefetch nodes in ***I*** and push nodes into ***N***  **while *N*** is not empty **do**  <node ***n***, ***dist***>= **Dequeue**(***N***)  **if**(***n*** is a leaf node) **then**   ***L*** = ***L*** ∪ {***n***}  **else**(***n*** is not a leaf node) **then**   **for**(each MBR ***m_i_*** in ***n***) **do** **if**(**mindist**(***m_i_***, ***q***) <= ***r***)    ***I*** = ***I*** ∪ {corresponding row key of ***m_i_***}    ObjectCounter+ = *number of objects for **n***    **if**(***ObjectCounter*** > ***k*** && ***r*** > **maxdist**(***m_i_***, ***q***))     update ***r*** to **maxdist**(***m_i_***, ***q***) ***L = L −*** {nodes in ***L*** located outside of range ***r***} ***D*** = {Prefetch objects in ***L***} /*Find candidate set of kNNs*/ **for** each object ***p*** in ***D* do**  **if**(**distance**(***p***, ***q***)**< = *r***)   **Enqueue**(***Q***, ***p***)   **if**(**size of**
***Q***
**>=**
***k***)    ***r*** = update to the distance to the farthest candidate  **else**   **break** **return *Q***

## 5. Performance Analysis

### 5.1. Experimental Setup and Datasets

For all experiments, we compared our method (labeled QbMBR tree in the graphs) with two other HBase table based indexing methods, MD-HBase [12] (labeled MD-HBase in the graphs) and KR+ index [13]. The average response time for 100 random queries was used for comparison. We set the parameters of MD-HBase and KR+ index for the experiments according to the analysis in the paper of KR+ index [13]. In their experimental analysis for threshold estimation, the capacity of the grid cell for MD-HBase was set to 2500. The parameters for KR+ indexing were the upper and lower bounds of the rectangles, and the order of the grid partitioning. We set the bounds of the rectangle to (100, 50) and the order to eight. The QbMBR tree used the capacity of the QbMBR as a parameter. In varying the capacity, there was a trade-off between the complexity of indexing and the selectivity.

We used two synthetically generated databases and two real databases for evaluation. The first synthetic database contained two-dimensional uniformly distributed data, and the second contained two-dimensional data that followed a normal distribution. The real datasets were the European POI dataset and the OpenStreetMap dataset. The European POI dataset contained one million objects, from which one million points were randomly sampled from a dataset at [18], and ten million points from the OpenStreetMap dataset were randomly sampled from a dataset at [19]. Our experiments were performed on an HBase cluster with four physical machines. The hardware specs for each machine were a 3.4 GHz i7-3770 processor, 8 GB RAM and 64-bit Linux operating system (version 16.04). We implemented our method using HBase 2.0 and Hadoop 2.7.4 as the underlying systems.

### 5.2. Performance Evaluation for Range Query

#### 5.2.1. Effect of Query Radius

**Figure 10:**
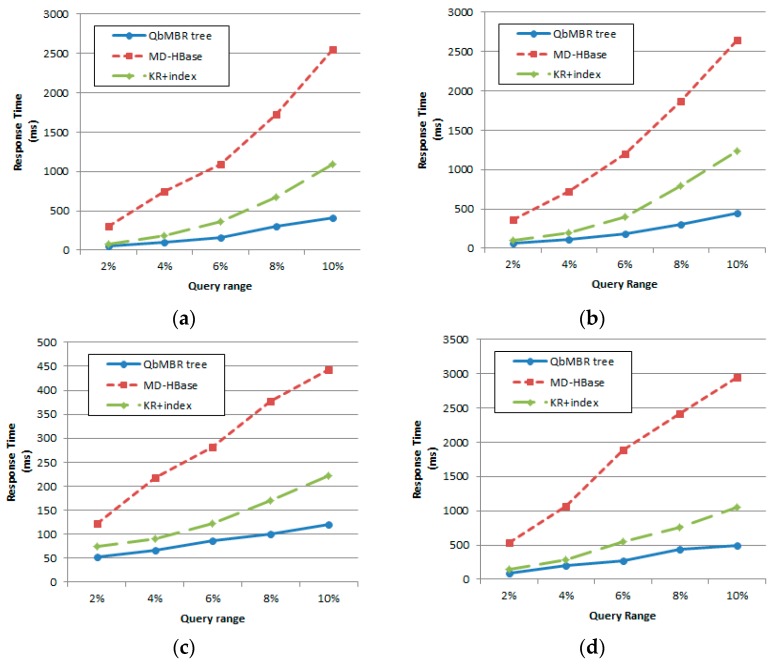
Effect of query radius on response time: (**a**) Synthetic dataset with uniform distribution; (**b**) Synthetic dataset with normal distribution; (**c**) European POI dataset; (**d**) OpenStreetMap dataset.

To evaluate the effects of query radius, the size of the synthetic database was fixed at 10 million points. Figure 10 plots the response times for range queries with query radii ranging from 1% to 5% of the data space. Note that the query range (i.e., query diameter) in Figure 10 is computed by multiplying 2 to a given query radius. As shown in Figure 10, the QbMBR tree outperformed MD-HBase and KR+ indexing. In particular, as the size of the retrieved dataset increased, the QbMBR tree achieved significantly better performance than the other two methods. For each query, we observed that the false positive rate of QbMBR was reduced compared to the other methods by 10–25%. Because the reduction of false positives leads to a decrease in RPCs, data loading, and computation time, the gap in performance was larger than the gap in the number of false positives. This tendency becomes increasingly apparent as more cells overlap as the query range increases. Although KR+ indexing groups data very precisely, it suffers from high numbers of false positives because its search process is based on grid partitioning.

#### 5.2.2. Effect of Database Size

**Figure 11:**
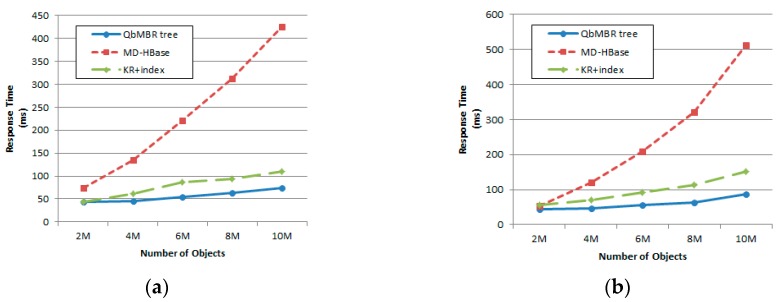
Effect of database size on response time: (**a**) Synthetic dataset with uniform distribution; (**b**) Synthetic dataset with normal distribution.

For this set of experiments, database size was increased from one million to 10 million points. The query radius was fixed at 2% of the data space. Figure 11 shows that as database size increased, the response time also increased for all methods. When the size of the database was relatively small, three of the methods achieved similar results. This is because if objects are sparsely distributed in space, the numbers of overlapping cells within the query range decreases. For example, only three or four cells are visited to answer the query in the case of a database with two million points. However, as can be seen from the figure, the rate of increase in response time for the QbMBR tree is lower than for the other two methods. Additionally, for KR+ indexing to yield optimal performance, we observe that it is necessary to change the threshold according to the distribution of the data. Because KR+ indexing partitions the data space into fixed-sized grid cells, it is relatively sensitive to data distributions.

### 5.3. Performance Evaluation for kNN Query

#### 5.3.1. Effect of the Number of Nearest Neighbors

**Figure 12:**
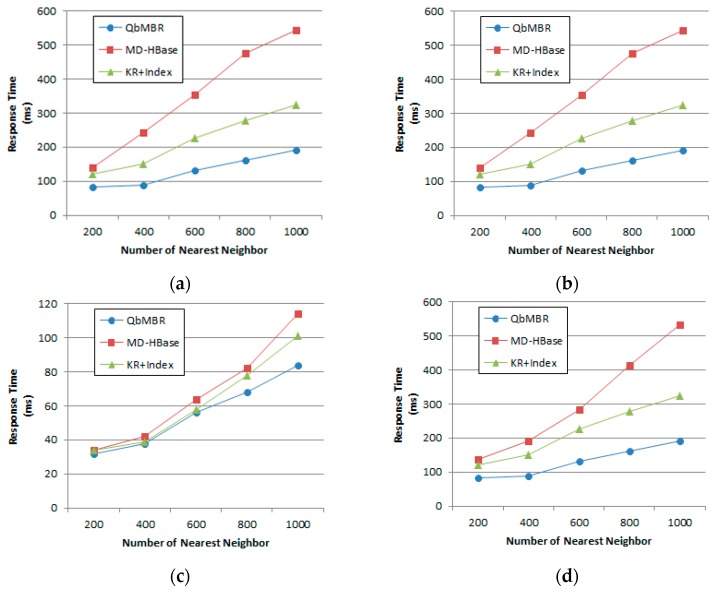
Effect of number of nearest neighbors on response time: (**a**) Synthetic dataset with uniform distribution; (**b**) Synthetic dataset with normal distribution; (**c**) European POI dataset; (**d**) Open street map dataset.

For this experiment, the size of the synthetic database was fixed at 10 million points. Figure 12 plots the response times for kNN queries with the number of nearest neighbors ranging from 200 to 1000. In this experiment, the QbMBR tree outperformed MD-HBase and KR+ indexing. The kNN query processing algorithms of the other two methods first pre-computed the grid cell that covered the query point. Because these algorithms can access the corresponding rows without any other search process, they showed better performance than our method when the database size or number of neighbors was small. However, as the parameter *k* increased, their performance degraded because retrieving a single cell was not sufficient to respond to the queries. If there were other grid cells located closer than the farthest candidate object, these algorithms repeatedly searched the index and objects until there were no more cells to be searched. Because this process caused a large number of RPCs and redundant scans on table, our method showed better performance when the value of *k* is increased.

#### 5.3.2. Effect of Database Size

**Figure 13:**
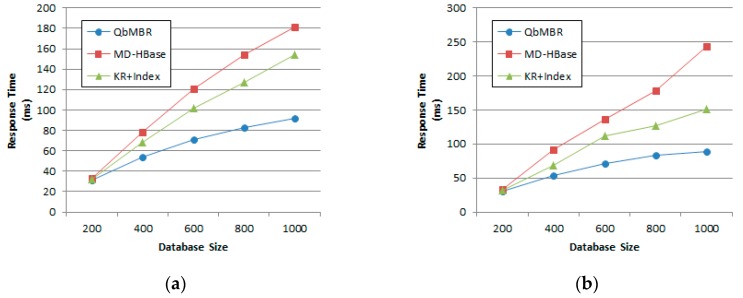
Effect of database size on response time: (**a**) Synthetic dataset with uniform distribution; (**b**) Synthetic dataset with normal distribution.

For this set of experiments, database size was increased from one million to 10 million points. The number of nearest neighbors was fixed at 400. Figure 13 shows that increasing database size decreases the performance for response time. As the number of data points increased, the size of the cells became smaller, and additional searches for adjacent cells occurred more frequently. For this reason, algorithms that had difficulty in extending their searches to adjacent cells suffered from increasing performance degradation as the size of the database increased.

### 5.4. Performance Evaluation for Index Construction and Update

**Figure 14:**
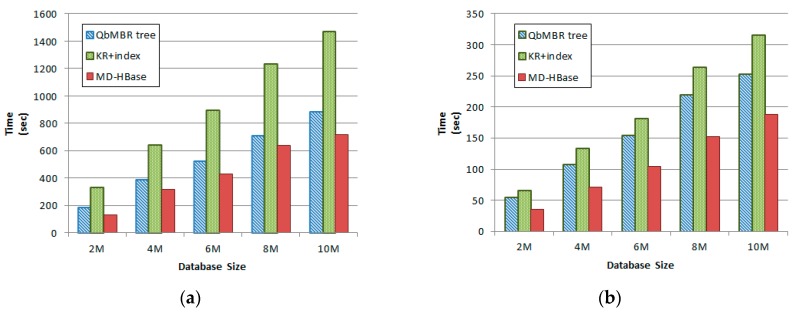
Time for index construction and update for different sized databases: (**a**) Time for index construction; (**b**) Time for incremental update of index.

To evaluate the cost of the index update, we performed two experiments using synthetically generated data. The first experiment evaluated the index construction time for the given number of objects. We increased the number of objects from two million to ten million and measured the index construction time. For the index construction of the databases, these methods exploit the memory to avoid the cost of object reinsertion for node splitting. Figure 14a shows the result of the experiment for construction. In this result, MD-HBase shows better performance than the other two methods. Our method uses a simple and fast node-splitting algorithm, whereas the KR+ index uses a more complex algorithm for R+ tree. In the case of MDHBase, the index construction algorithm is almost same to kd-tree.

In the second experiment, the incremental update cost for the index stored in the table was measured. In this experiment, we measured the incremental index update time by increasing each database size by 1%. Figure 14b shows the result of the update experiment. When updating the index, MD-HBase showed a superior performance than the other two methods. In this method, the row key of an inserted object was computed in less time, and a reinsertion was not required even when the index was updated. Compared to HBase, our method showed an overhead in which objects of a split node were re-inserted. As a result, the time required for the update was increased by about 30%. KR+ index required more time because it showed the overhead for object reinsertion and a more complex node-splitting algorithm of the R+ tree.

### 5.5. Effect of HBase Cluster Sizes

**Figure 15:**
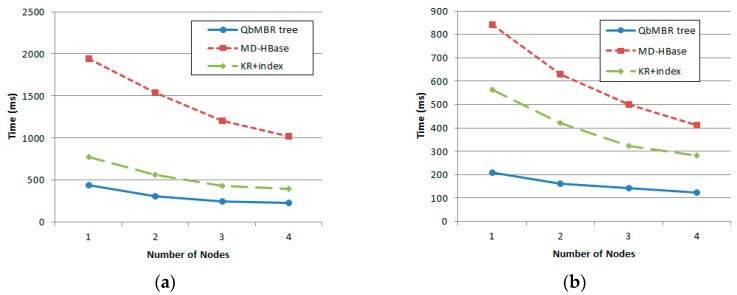
Effect of cluster size on response time: (**a**) Response time of range query; (**b**) Response time of kNN query.

In this experiment, we evaluated the scalability of HBase clusters by varying the cluster size from 1 to 4. Figure 15 plots the response times of range queries and kNN queries on the different cluster sizes. The radius of range queries was fixed to 3% of the data space and the number of nearest neighbors of kNN queries was fixed to 800. The synthetic database with 10 million points was used in this experiment.

As shown in Figure 15a, increasing number of nodes in HBase cluster improves the performance of the range queries for all three methods. In particular, the performance improvement of MD-HBase was more significant compared to QbMBR tree and KR+ index as the cluster size was increased. The reason is that the increasing number of nodes in the cluster significantly reduces the I/O cost of the *scan* operations for a large number of false positives occurred in MD-HBase. Since QbMBR tree produces much less number of false positives than MD-HBase and KR+ index, its I/O cost improvement is not as much as MD-HBase. However, QbMBR tree always fetches the fewer objects than MD-HBase and KR+ index. Therefore, its performance constantly remains better than the other two methods regardless of cluster size.

In the case of kNN query shown in Figure 15b, our method also shows the better performance with increasing size of cluster. As discussed in Section 5.3.1, MD-HBase and KR+ index require additional I/O costs if kNN candidates are not located in a single grid cell. Since increasing number of nodes in cluster can help to process this task more efficiently, the performance improvements of MD-HBase and KR+ index are more significant than QbMBR tree.

## 6. Conclusions

This paper presented the QbMBR tree, which is an efficient indexing scheme for handling big spatial data in HBase. The proposed scheme recursively divides the data space into quadrants and creates MBRs for each quadrant to build a hierarchical indexing structure. QbMBR provided better filtering power for processing spatial queries when compared to existing schemes. Our proposed method minimized access to areas that were not relevant, thereby reducing the amount of data that had to be transmitted and the number of expensive RPCs during query processing. Additionally, our method reduced the total size of the database because the lengths of the row keys used to store the data were shorter. The algorithms for range query and kNN query using the QbMBR tree were also presented in this paper. Our proposed algorithms significantly reduced query response times by prefetching the necessary index nodes and data into memory while traversing the QbMBR tree. Experimental results demonstrated that our proposed algorithms outperformed two existing methods: MD-HBase and KR+ indexing.

In the Internet of Everything (IoE) environment, a vast amount of sensor data, such as temperature, humidity, light, and sound, are generated with their location information by dynamic sensor devices. The locations and proximities of dynamic sensors are fundamental information used in various services and applications. Our method can provide efficient databases to store and search the location of dynamically updated sensor devices and their sensor data.

## References

[1] Lee J.-G., Kang M. (2015). Geospatial Big Data: Challenges and Opportunities. Big Data Res..

[2] Eldawy A., Mokbel M.F. The Era of Big Spatial Data: A Survey. Proceedings of the IEEE 31st International Conference on Data Engineering Workshops.

[3] Cary A., Sun Z., Hristidisand V., Rishe N. (2009). Experiences on Processing Spatial Data with MapReduce. Lecture Notes in Computer Science Scientific and Statistical Database Management.

[4] Wang K., Han J., Tu B., Dai J., Zhou W., Song X. Accelerating Spatial Data Processing with MapReduce. Proceedings of the IEEE 16th International Conference on Parallel and Distributed Systems.

[5] Zhang S., Han J., Liu Z., Wang K., Feng S. Spatial Queries Evaluation with MapReduce. Proceedings of the Eighth International Conference on Grid and Cooperative Computing.

[6] Eldawy A., Mokbel M.F. SpatialHadoop: A MapReduce framework for spatial data. Proceedings of the IEEE 31st International Conference on Data Engineering.

[7] Aji A., Wang F., Vo H., Lee R., Liu Q., Zhang X., Saltz J. (2013). Hadoop GIS. A High Performance Spatial Data Warehousing System over MapReduce. Proc. VLDB Endow..

[8] Dimiduk N., Khurana A. (2013). HBase in Action.

[9] Lee K., Ganti R.K., Srivatsa M., Liu L. Efficient Spatial Query Processing for Big Data. Proceedings of the 22nd ACM SIGSPATIAL International Conference on Advances in Geographic Information Systems.

[10] Huang S., Wang B., Zhu J., Wang G., Yu G. R-HBASE: A Multi-dimensional Indexing Framework for Cloud Computing Environment. Proceedings of the IEEE International Conference on Data Mining Workshop.

[11] Van L.H., Takasu A. An Efficient Distributed Index for Geospatial Databases. Proceedings of the International Conference on Database and Expert Systems Applications.

[12] Nishimura S., Das S., Agrawal D., Abbadi A.E. (2012). MD-HBase: Design and implementation of an elastic data infrastructure for cloud-scale location services. Distrib. Parallel Databases.

[13] Wei L., Hsu Y., Peng W., Lee W. (2014). Indexing spatial data in cloud data managements. Pervasive Mob. Comput..

[14] Jo B., Jung S. (2018). Quadrant-Based MBR-Tree Indexing Technique for Range Query Over HBase. Proceedings of the 7th International Conference on Emerging Databases.

[15] Wang L., Chen B., Liu Y. Distributed storage and index of vector spatial data based on HBase. Proceedings of the 21th International Conference on Geoinformatics.

[16] Li S., Hu S. Pyro: A Spatial-Temporal Big-Data Storage System. Proceedings of the ACM USENIX Annual Technical Conference.

[17] Chen X., Zhang C., Ge B., Xiao W. Spatio-temporal Queries in HBase. Proceedings of the IEEE International Conference on Big Data.

[18] PocketGPSWorld. https://www.pocketgpsworld.com/.

[19] OpenStreetMap. https://planet.openstreetmap.org/.

